# Cardiac Organoids Uncover an Association of CHD7 Deficiency with Congenital Heart Disease

**DOI:** 10.1007/s43657-026-00319-z

**Published:** 2026-07-09

**Authors:** Siyu Sun, Zhiyu Feng, Weilan Na, Yuan Gao, Shaojie Min, Quannan Zhuang, Siyi Lin, Quming Zhao, Xianghui Huang, Hongmei Song, Wei Sheng, Guoying Huang

**Affiliations:** 1 Chinese Academy of Medical Sciences and Peking Union Medical College, Peking Union Medical College Hospital, Department of Pediatrics, Research Unit of Early Intervention of Genetically Related Childhood Cardiovascular Diseases (2018RU002), Children’s Hospital of Fudan University, Shanghai, 201102 China; 2 Pediatric Heart Center, Children’s Hospital of Fudan University, Shanghai, 201102 China; 3 Shanghai Key Laboratory of Birth Defects, Children’s Hospital of Fudan University, Shanghai, 201002 China; 4 Fujian Key Laboratory of Neonatal Diseases, Xiamen Children’s Hospital, Fujian, 361006 China; 5 Department of Pediatrics, Peking Union Medical College Hospital, Chinese Academy of Medical Sciences and Peking Union Medical College, Beijing, 100730 China

**Keywords:** Cardiac organoids, CHD7, Congenital heart disease, CHARGE syndrome

## Abstract

CHD7 (Chromodomain helicase DNA-binding protein 7) encodes an ATP-dependent chromatin remodeler, and pathogenic disruption of CHD7 underlies CHARGE syndrome(Coloboma, Heart defects, Atresia choanae, Retarded growth and development, Genital abnormalities, Ear abnormalities syndrome), a congenital disorder frequently accompanied by complex cardiac malformations. However, how CHD7 contributes to human cardiogenesis remains poorly defined. Here, we use multi-chamber cardiac organoids derived from human embryonic stem cells, which capture key features of early cardiac morphogenesis and regional lineage patterning, to interrogate CHD7 function. CHD7 loss leads to attenuation of cardiac progenitor gene programs, disrupted chamber organization, reduced efficiency of cardiomyocyte differentiation, and markedly impaired contractile function. Bulk RNA sequencing further reveals broad misregulation of transcriptional modules involved in cardiogenic signaling, regional progenitor patterning, and myocardial maturation. Together, these data establish a human organoid framework for modelling CHD7-associated congenital heart disease and identify CHD7 as a key regulator of cardiac morphogenesis and the acquisition of functional cardiomyocyte properties.

**Graphical Abstract:**

**Figure:**
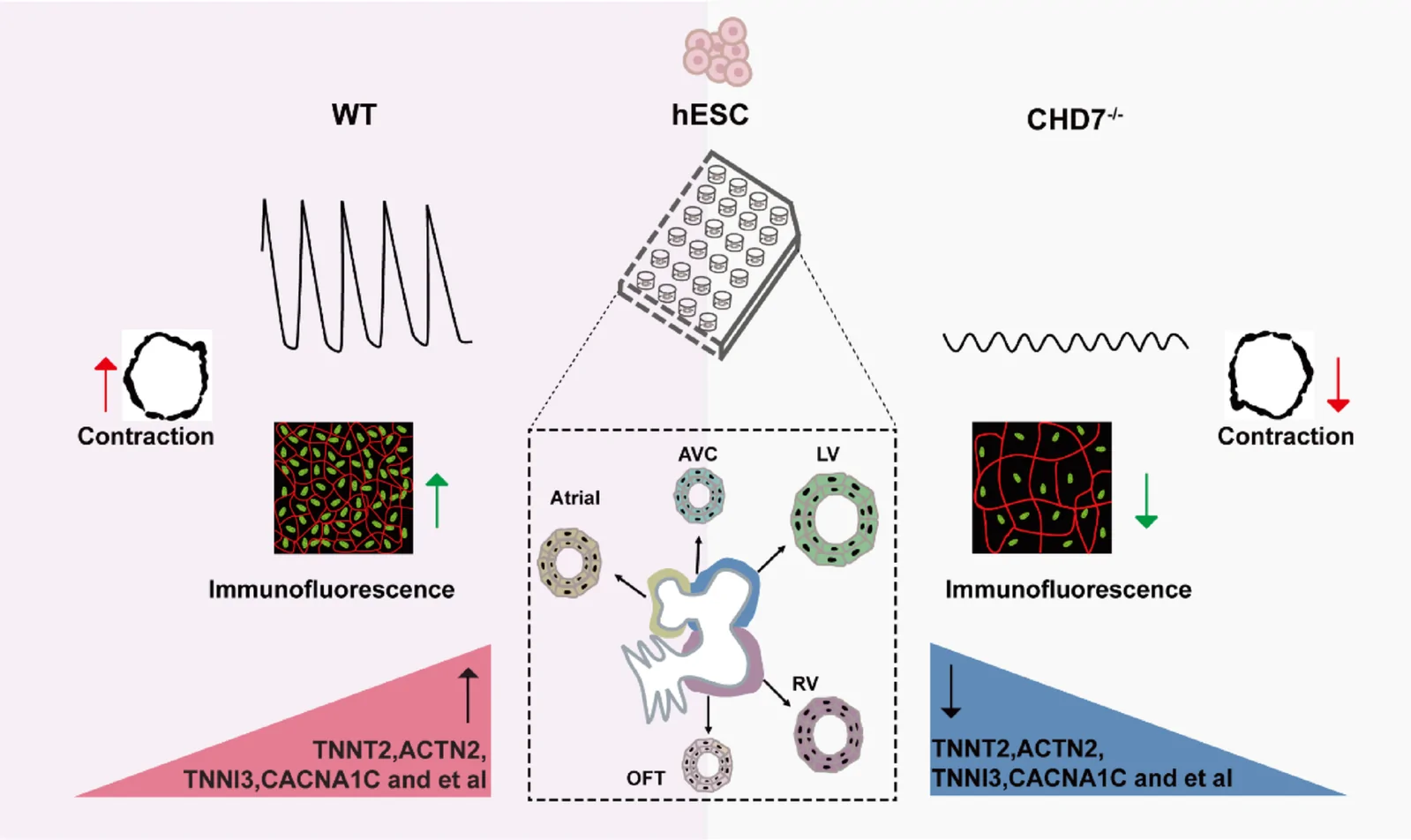

**Supplementary Information:**

The online version contains supplementary material available at https://doi.org/10.1007/s43657-026-00319-z.

## Introduction

CHARGE syndrome is a rare multisystem developmental disorder with an estimated incidence of approximately 1 in 10,000 live births (Issekutz et al. 2005; Jyonouchi et al. 2009; van Ravenswaaij-Arts and Martin 2017). Clinically, it is characterised by a stereotyped constellation of anomalies, including ocular coloboma (Krueger and Morris 2022), choanal atresia (Bergman et al. 2011), growth retardation (Breuer et al. 2024; Dauber et al. 2010; Huang et al. 2025; Jamadagni et al. 2021; Kim and Roberts 2013; Liu et al. 2022), genital hypoplasia (Antoniou et al. 2019; Dauber et al. 2010), ear abnormalities (Gao et al. 2024; Nie et al. 2022; Yao et al. 2018), and congenital heart defects (van Ravenswaaij-Arts and Martin 2017; Vissers et al. 2004). . Cardiac involvement is a major driver of morbidity and early mortality in affected children. Approximately three-quarters of individuals with CHARGE syndrome present with structural heart malformations, most commonly atrioventricular septal defects and outflow tract abnormalities, including aortic arch anomalies (Bergman et al. 2011; Yan et al. 2020).

Chromodomain helicase DNA-binding protein 7 (CHD7) encodes an ATP-dependent chromatin remodeler with broad roles across embryonic development (van Ravenswaaij-Arts and Martin 2017; Wang et al. 2025), and *de novo* pathogenic variants in *CHD7* account for the majority of CHARGE syndrome cases (Gug et al. 2020; Huang et al. 2023; Wang et al. 2022; Zhang et al. 2024). Notably, our previous work identified *CHD7* loss-of-function variants in sporadic congenital heart disease, suggesting that CHD7-related cardiac pathology may extend beyond the syndromic context (Huang et al. 2023). *In vivo* studies further support an essential role for CHD7 in cardiogenesis: global *Chd7* inactivation in mice causes early embryonic lethality (Hurd et al. 2007), and conditional deletion in cardiac neural crest cells (*Wnt1*-Cre) (Yan et al. 2020) or cardiogenic mesoderm (*Mesp1*-Cre) (Stathopoulou et al. 2023) results in severe cardiac malformations or embryonic demise. However, the early lethality of many *Chd7* loss-of-function models limits mechanistic dissection of CHD7 function during later stages of chamber formation and cardiomyocyte differentiation, and hampers the resolution of cell-type-specific requirements and regulatory programs.

Human embryonic stem cells (hESCs) provide a tractable platform to model human cardiogenesis *in vitro*. Under defined conditions, hESCs can be directed to self-organise into cardiac organoids that capture key aspects of early morphogenesis and multicellular patterning, while reproducing features relevant to congenital heart disease (Hofbauer et al. 2021; Lewis-Israeli et al. 2021; Mendjan et al. 2025; Schmidt et al. 2023). In particular, stage-controlled differentiation protocols enable the generation of multi-chamber cardiac organoids with chamber-like architecture and regionally patterned cardiac lineages, offering an experimentally controllable system in which developmental trajectories and gene-regulatory perturbations can be interrogated in a human context (Kim et al. 2022; Lewis-Israeli et al. 2021; Schmidt et al. 2023; Srivastava 2021).

Here, we generated multi-chamber cardiac organoids from *CHD7*-knockout hESCs and combined phenotypic analysis with endothelial differentiation assays and integrative transcriptomic and epigenomic profiling to define the consequences of CHD7 loss during human heart development. Our results show that CHD7 is required for proper organoid morphogenesis and efficient cardiomyocyte differentiation, providing mechanistic insight into CHD7-associated congenital heart disease.

## Materials and Methods

### HESC Culture and Cardioid Generation

#### HESC Culture

Human embryonic stem cells (hESCs) were maintained under feeder-free conditions on Matrigel-coated plates (Corning, 356255) in mTeSR™ Plus medium (Stemcell Technologies, 100–0276). Cultures were passaged at approximately 80% confluence using Accutase (Sigma, A6964) and replated at a 1:5 ratio. The medium was refreshed daily, and only early-passage, morphologically uniform colonies were used for differentiation experiments.

### Cardiac Organoids Differentiation

Cardiac organoid formation was performed following a modified version of the protocol described by Schmidt et al. (2023). Briefly, hESCs were seeded into 24-well plates (Corning, 353047) at a density of 3–4 × 10⁴ cells per well in mTeSR™ Plus medium supplemented with the ROCK inhibitor Y-27632 (5 µM, Selleck, S1049). All differentiation media were prepared using a chemically defined medium (CDM) composed of 50% IMDM (Gibco, 21980065) and 50% F12 Nutrient Mix (Gibco, 31765068), supplemented with 5 mg/mL bovine serum albumin (BSA, Sigma, V900933), 1% concentrated lipids (Gibco, 11905031), 0.004% monothioglycerol (MCE, HY-W010572), and 15 µg/mL transferrin (MCE, HY-P3267).

After 24 h, the culture medium was replaced with mesoderm induction medium, consisting of CDM supplemented with FLYAB, including FGF2 (R&D Systems, 3718-FB), LY294002 (MCE, HY-10108), Activin A (R&D Systems, 338-AC), BMP4 (R&D Systems, 314-BP-050), CHIR99021 (Selleck, S1263).

After 36–40 h of induction (Table 1, day 0–1.5), cells were dissociated with TrypLE™ Express (Gibco, 12605010) and transferred into ultra-low attachment 96-well plates (Corning, 7007) at 1.5 × 10⁴ cells per well in cardiac mesoderm patterning medium. The plates were centrifuged at 200 × *g* for 5 min. The culture medium was replaced with cardiac mesoderm patterning media 1 (Table 2, day 1.5–3.5), consisting of CDM supplemented with BMP4, FGF2, insulin (MCE, HY-P73243), either Wnt-C59 (C59, MCE, HY-15659) or XAV-939 (MCE, HY-15147), and retinoic acid (MCE, HY-14649). For the subsequent two days of culture (Table 3, day 3.5–5.5), media were exchanged every other day with cardiac mesoderm patterning media 2 containing BMP4, FGF2, insulin, either C59 or XAV-939, and retinoic acid. For the subsequent 48 h of culture (Table 4, day 5.5–7.5), media were exchanged every other day with cardiomyocyte specification media containing BMP4 (10 ng/ml), FGF2 (8 ng/ml), and insulin (10 µg/ml), with concentrations for each subtype shown below.

**Table 1:** Mesoderm Induction Media for all cell lines unless otherwise specified (day 0–1.5)

Subtype	Time (day)	Concentrations
LV (FHF-derived)	0–1.5	BMP4 (10 ng/ml), FGF2 (30 ng/ml), LY294002 (5 µM), Activin A (5 ng/ml), and CHIR99021 (1 µM)
RV (aSHF-derived)	0–1.5	BMP4 (10 ng/ml), FGF2 (30 ng/ml), LY294002 (5 µM), Activin A (50 ng/ml), and CHIR99021 (1.5 µM)
OFT	0–1.5	BMP4 (10 ng/ml), FGF2 (30 ng/ml), LY294002 (5 µM), Activin A (50 ng/ml), and CHIR99021 (1.5 µM)
Atria (pSHF-derived)	0–1.5	BMP4 (10 ng/ml), FGF2 (30 ng/ml), LY294002 (5 µM), Activin A (10 ng/ml), and CHIR99021 (2 µM)
AVC	0–1.5	BMP4 (10 ng/ml), FGF2 (30 ng/ml), LY294002 (5 µM), Activin A (10 ng/ml), and CHIR99021 (2 µM)

**Table 2:** Cardiac Mesoderm Patterning Media 1 (day 1.5–3.5)

Subtype	Time (day)	Concentrations
LV (FHF-derived)	1.5–3.5	BMP4 (10 ng/ml), FGF2 (8 ng/ml), insulin (10 µg/ml), C59 (2 µM) or XAV-939 (5 µM), and retinoic acid (50 nM)
RV (aSHF-derived)	1.5–3.5	TGF-β inhibitor SB 431542 (10 µM), C59 (2 µM) or XAV-939 (5 µM)
OFT	1.5–3.5	TGF-β inhibitor SB 431542 (10 µM), C59 (2 µM) or XAV-939 (5 µM)
Atria (pSHF-derived)	1.5–3.5	SB 431542 (10 µM), C59 (2 µM) or XAV-939 (5 µM)
AVC	1.5–3.5	BMP4 (10 ng/ml), SB 431542 (10 µM), C59 (2 µM) or XAV-939 (5 µM), and retinoic acid (500 nM)

**Table 3:** Cardiac Mesoderm Patterning Media 2 (day 3.5–5.5)

Subtype	Time (day)	Concentrations
LV (FHF-derived)	3.5–5.5	BMP4 (10 ng/ml), FGF2 (8 ng/ml), insulin (10 µg/ml), C59 (2 µM) or XAV-939 (5 µM), and retinoic acid (50 nM)
RV (aSHF-derived)	3.5–5.5	BMP4 (10 ng/ml), FGF2 (8 ng/ml), insulin (10 µg/ml), C59 (2 µM) or XAV-939 (5 µM), and retinoic acid (500 nM)
OFT	3.5–5.5	BMP4 (10 ng/ml), FGF2 (8 ng/ml), insulin (10 µg/ml), and C59 (2 µM) or XAV-939 (5 µM)
Atria (pSHF-derived)	3.5–5.5	BMP4 (10 ng/ml), FGF2 (8 ng/ml), insulin (10 µg/ml), C59 (2 µM) or XAV-939 (5 µM), and retinoic acid (500 nM)
AVC	3.5–5.5	BMP4 (10 ng/ml), FGF2 (8 ng/ml), insulin (10 µg/ml), XAV-939 (5 µM), and retinoic acid (500 nM)

**Table 4:** Cardiomyocyte Specification Media (day 5.5–7.5)

Subtype	Time (day)	Concentrations
LV (FHF-derived)	5.5–7.5	BMP4 (10 ng/ml), FGF2 (8 ng/ml), and insulin (10 µg/ml)
RV (aSHF-derived)	5.5–7.5	BMP4 (10 ng/ml), FGF2 (8 ng/ml), and insulin (10 µg/ml)
OFT	5.5–7.5	BMP4 (10 ng/ml), FGF2 (8 ng/ml), and insulin (10 µg/ml)
Atria (pSHF-derived)	5.5–7.5	BMP4 (10 ng/ml), FGF2 (8 ng/ml), and insulin (10 µg/ml)
AVC	5.5–7.5	BMP4 (10 ng/ml), FGF2 (8 ng/ml), and insulin (10 µg/ml)

For the subsequent days of culture, media were exchanged every other day with CDM containing insulin (10 µg/ml).

### 2D Endothelial Cell Differentiation

HESCs were seeded at a density of 100,000 cells per vitronectin-coated 24-well plate in mTeSR™ Plus medium supplemented with Y-27632 (5 µM). After 24 h, differentiation was initiated with FLYAB medium containing FGF2 (30 ng/ml), LY294002 (5 µM), Activin A (50 ng/ml), BMP4 (10 ng/ml), and CHIR99021 at 1 µMfor FHF or 1.5 µM for aSHF and pSHF induction, followed by incubation for 36–40 h. Subsequently, cells were cultured for 2 days in the corresponding Cardiac Mesoderm Patterning Medium 1 specific to each subtype. Endothelial specification was then induced by culturing the cells in CDM supplemented with VEGF (200 ng/ml, Peprotech, AF-100-20) and Forskolin (2 µM, MCE, HY-15371) for 2 days, followed by CDM containing VEGF (100 ng/ml) for an additional day.

### Generation of *CHD*7-knockout hESC Cell Lines

*CHD7*-knockout hESC lines were generated using the epiCRISPR/Cas9 genome editing system. Guide RNAs (gRNAs) were designed using DeepHF (https://www.deephf.com/) and cloned into the epiCRISPR vector (a kind gift from Dr. Yongming Wang). The resulting constructs were transfected into hESCs using the Lonza 4D Nucleofector system (Lonza, AAF-1002X). Following puromycin selection (Thermo, A1113803) for one week, single clones were screened by genomic DNA sequencing and confirmed by Western blotting.

### Reverse Transcription–Quantitative Real-Time Polymerase Chain Reaction (RT-qPCR)

Total RNA was extracted using TRIZOL reagent (Thermo, 15596018) following the manufacturer's protocol. RNA (1 µg) was reverse-transcribed into cDNA using the PrimeScript™ RT reagent Kit (Takara, RR036A). RT-qPCR was performed using the TB Green^®^ Premix Ex Taq™ (Takara, RR420A) on a QuantStudio System (Thermo Fisher Scientific). Gene expression levels were normalized to internal controls, and relative expression was determined using the _ΔΔ_Ct method. Primers are listed in Supplementary Table 1.

### Immunofluorescence Staining

HESCs were separated into single-cell suspensions using Accutase (Sigma, A6964) and plated at a 1:10 ratio on 15-mm Matrigel-coated dishes for immunofluorescence labeling. Cells were cultured to the appropriate confluence, fixed in 4% paraformaldehyde (PFA, Solarbio, #P1110) for 20 min at room temperature, permeabilized with 0.2% Triton X-100 for another 20 min, blocked with 5% BSA for 1 h before staining, and then washed three times with PBS (Solarbio, P1020). After three PBS washes, samples were incubated with primary antibodies against OCT4 (Abcam, ab181557, 1:300), SSEA4 (Abcam, ab16287, 1:300), cardiac troponin T (TNNT2; Thermo Fisher Scientific, MA5-12960, 1:200), CD31 (Abcam, ab9498, 1:300), and ACTA2 (Proteintech, ab124964, 1:600) overnight at 4 °C. Following washing, cells were incubated with Alexa Fluor-conjugated secondary antibodies (Thermo, A32731 and A32723) for 1 h at room temperature. Nuclei were counterstained with fluorescence mounting medium (Beyotime, DUO82040) for 15 min.

### Cryosectioning

Cardiac organoids were fixed in 4% PFA for 20 min, rinsed with PBS, and immersed in 30% sucrose solution for cryoprotection. Samples were embedded in OCT compound, rapidly frozen on a pre-chilled metal block submerged in liquid nitrogen, and stored at − 80 °C until sectioning. Cryosections were cut using a Leica cryostat, mounted onto SuperFrost Plus slides, and stored at − 20 °C to − 80 °C prior to immunostaining.

### Immunostaining

To remove OCT, fixed specimens were washed in PBS for 15 min. Optionally, samples were permeabilized in 0.2% Triton X-100 for 15 min to enhance antibody penetration. Blocking was performed for at least 30 min in PBS containing 5% BSA and 0.2% Triton X-100. Sections were then incubated overnight at 4 °C with primary antibodies diluted in 5% BSA. The next day, slides were washed three times in PBS containing 0.1% Tween-20 (Sigma, P1379), followed by incubation for 1 h at room temperature in 5% BSA containing the secondary antibody. Finally, samples were washed in PBS with 0.1% Tween-20. Slides were mounted using a fluorescence mounting medium (Beyotime, DUO82040) and covered with a coverslip.

### Calcium Transient Imaging

On day 9.5(D9.5) of differentiation, cardiac organoids were incubated with 5 µM Cal-520^®^ AM (AAT Bioquest, 21130) and 0.02% Pluronic F-127 (AAT Bioquest, 20053) in Tyrode’s solution (Solarbio, T1420) at 37 °C for 30 min. Following dye loading, organoids were rinsed three times with Tyrode's solution to remove excess dye. Calcium transients were recorded using a Leica DMi8 fluorescence microscope at a temporal resolution of 10 ms per scan over 30 s. Recorded data were subsequently analyzed and visualized using ImageJ/Fiji.

### MuscleMotion Contraction Analysis

Contractile activity of WT and *CHD7*^−/−^ hESC-derived cardiac organoids was assessed between D7.5 and D9.5 of differentiation. Time-lapse videos were acquired using a Leica inverted microscope equipped with a 10× objective, with each recording lasting 20–30 s. For each condition, three videos were collected per well from three to five wells across three independent differentiation batches. The recordings were converted to TIFF image sequences at 9.97 frames per second using FFmpeg (version 8.0) and subsequently compiled into stacked TIFF files for analysis with the MuscleMotion macro in ImageJ/Fiji (Patel et al. 2024; Sala et al. 2018). Quantitative parameters extracted included contraction amplitude, time to peak, relaxation time, and interbeat interval. To minimize environmental variability, organoids were kept outside the incubator for no longer than 5 min during imaging and immediately returned to temperature-controlled conditions to preserve contractile activity.

### Western Blotting Analysis

Total protein extracts were prepared from cells using RIPA lysis buffer (Solarbio, R0010) supplemented with a protease inhibitor cocktail. Samples were mixed with 1× sample buffer (GenScript, M01015), boiled for 5 min, and resolved on 4–20% SurePAGE™ Bis-Tris gels (GenScript, M00655). Proteins were then transferred to PVDF membranes (Millipore, IPVH00010).

Membranes were blocked in TBST (Tris-buffered saline with 0.1% Tween-20) containing 5% non-fat dry milk (BD Biosciences, 232100) for 1 h at room temperature, followed by overnight incubation at 4 °C with primary antibodies: CHD7 (Abcam, ab307499, 1:1000) and GAPDH (Proteintech, 10494-1-AP, 1:10,000). After three washes in TBST (15 min each), membranes were incubated with HRP-conjugated secondary antibodies (CST, 7074/7076, 1:4000) for 2 h at room temperature. Protein bands were developed using SuperSignal West Pico PLUS (Thermo Fisher Scientific, 34580) for 2 min and visualized on a ChemiDoc Imaging System (Bio-Rad XRS+). Band intensities were quantified using Image Lab software.

### Flow Cytometry

On D9.5 of differentiation, cardiac organoids were dissociated into single-cell suspensions using TrypLE Select (Thermo, 12563029) at 37 °C for 10 min. The cells were then washed with PBS containing 2% fetal bovine serum (FBS) and subsequently fixed in 4% PFA for 15 min at room temperature. For intracellular marker staining, fixed cells were incubated for 1 h at room temperature with primary antibodies against CD31 (Abcam, ab9498). After washing, samples were incubated for 30 min in the dark with the appropriate PE-conjugated secondary antibody . Finally, stained cells were analyzed on a BD flow cytometer, and data were processed and visualized using FlowJo.

### RNA Sequencing and Analysis

RNA-seq library preparation was conducted in accordance with the guidelines provided by the manufacturer (Vazyme, TR503-01). Libraries were subjected to high-throughput sequencing by the Novogene Company using Illumina NovaSeq 6000 platform (Beijing, China) with three biological replicates. The FASTQ data underwent adapter trimming using trim_galore (v0.6.4), and reads were mapped to the human genome reference (UCSC hg38) using Tophat software (Trapnell et al. 2009). The Cufflinks program (v2.2.1) was applied to allocate the mapped reads to human transcripts (UCSC hg38) using default settings. This process was to determine gene expression levels, which are represented by FPKM. Differentially expressed genes were determined using DESeq2 (Love et al. 2014) and defined by more than 1.5-fold change in FPKM and a *p*-value below 0.05.

### Statistical Analysis

All quantitative data are expressed as mean ± standard error of the mean (SEM), based on at least three independent biological replicates. For all experiments, "*n*"represents the number of independent biological replicates. Statistical comparisons between two groups were performed using the unpaired Student’s *t*-test, while categorical data were analyzed using the two-tailed Fisher’s exact test. A *p*-value < 0.05 was regarded as statistically significant. Graphs and statistical analyses were prepared using GraphPad Prism software (GraphPad Software 8.0).

## Results

### CHD7 Shows High and Broad Expression in Early-Stage Human and Mouse Hearts

To assess the developmental expression landscape of CHD7, we first analyzed transcriptomic profiles across human fetal tissues at 10 weeks of gestation using GSE64283. Consistent with *CHD7* being a developmentally regulated and broadly expressed gene, *CHD7* transcripts were detected across multiple tissues; however, expression was comparatively higher in the heart than in most other fetal tissues examined, including adrenal gland, intestine, kidney, lung, and stomach (Fig. S1a). We next examined the temporal dynamics of CHD7 expression during human cardiac development using GSE156707, which revealed that *CHD7* expression peaks around the critical window of human cardiac morphogenesis (approximately 10 weeks) and then declines across later embryonic stages (Fig. S1b). A similar pattern was observed in mouse, where analysis of GSE51483 showed elevated *Chd7* expression during early cardiogenesis (E7.5–E12.5) followed by a decrease at later stages (Fig. S1c), supporting an evolutionarily conserved enrichment of CHD7 during early heart development. Finally, single-nucleus RNA-seq data from human fetal hearts (GSE156707) indicated broad CHD7 expression across cardiac cell types, with highest expression in cardiomyocytes, followed by endothelial cells and additional non-myocyte populations (Fig. S1d–f). Together, these analyses demonstrate that CHD7 is highly expressed during early cardiogenesis in both human and mouse, supporting a potential role in cardiac morphogenesis and CHARGE-associated congenital heart disease.

### Establishment of CHD7-knockout Cardiac Organoids Reveals Early Defects in Regional Progenitor Programs and Chamber Morphogenesis

**Fig. 1:**
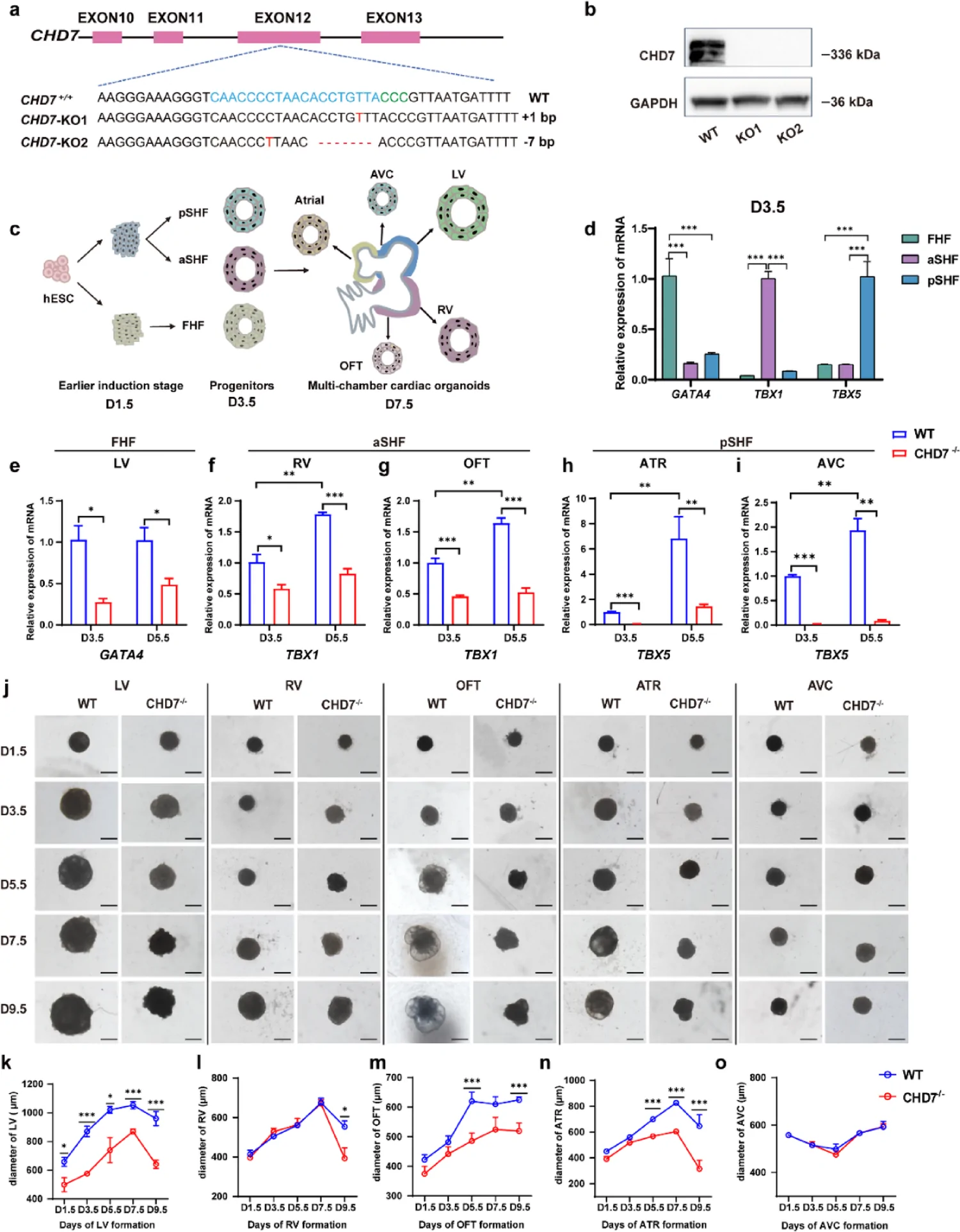
Generation and characterization of CHD7 knockout multi-chamber cardiac organoids. (**a**) *CHD7*^−/−^ hESC genome sequencing validation. (**b**) Western blotting assay verified the knockout of CHD7. (**c**) Schematic of the protocol for differentiation from hESCs to multi-chamber cardiac organoids. (**d**) RT-qPCR showing the relative expression of *GATA4*, *TBX1,* and *TBX5* of cardiac organoids at D3.5 (*n* = 3). (**e**) RT-qPCR showing the relative expression of *GATA4* of LV at D3.5 and D5.5 (*n* = 3). (**f**-**g**) RT-qPCR showing the relative expression of *TBX1* of RV and OFT at D3.5 and D5.5 (*n* = 3). (**h**-**i**) RT-qPCR showing the relative expression of *TBX5* of ATR and AVC at D3.5 and D5.5 (*n* = 3). (**j**) The development of multi-chamber cardiac organoids from D1.5 until D9.5 of WT and *CHD7*^−/−^ differentiation, scale bars = 50 μm. (**k**-**o**) Quantification of diameter of multi-chamber cardiac organoids from D1.5 until D9.5 (*n* ≥ 32). Organoid size was quantified by measuring diameter, with the narrowest dimension used for irregularly shaped organoids after cavity formation. Data are shown as means ± SEM. ns *p* > 0.05; *p < 0.05, **p < 0.01, ***p < 0.001

Immunofluorescence analysis confirmed detectable CHD7 expression across organoid-derived cardiac subtypes, consistent with a broad role for CHD7 during multi-chamber differentiation (Fig. S2a). The capabilities of DNA editing have been greatly expanded by advancements in CRISPR technologies, which have also improved the accuracy and efficiency of these methods. When combined, these advancements have great potential for use in cardiac development (Althali and Hentges 2022; Feng et al. 2024). To interrogate CHD7 function in human cardiogenesis, we generated *CHD7* knockout (*CHD*7^⁻/⁻^) hESC lines using CRISPR/Cas9 with a guide RNA targeting exon 12 (Fig. 1a). Efficient gene disruption was validated by western blotting (Fig. 1b).

To accurately delineate the role of CHD7 in cardiac development, we employed a multi-chamber cardiac organoid model capable of distinguishing distinct cardiac regions, including the left ventricle (LV), right ventricle (RV), atrium (ATR), outflow tract (OFT), and atrioventricular canal (AVC) (Schmidt et al. 2023). Among these, the LV is derived from the first heart field (FHF), whereas the RV and the majority of the OFT originate from the anterior second heart field (aSHF) and the ATR and part of the AVC arise from the posterior SHF (pSHF) (Fig. 1c). By stage-specific modulation of WNT and Activin/Nodal signaling, we robustly directed hESCs into regionally distinct cardiac organoids.

To define the earliest stage at which progenitor program defects emerge, we quantified lineage-restricted progenitor markers during the early differentiation window. At D 3.5, *GATA4* was enriched in FHF-associated progenitors relative to aSHF and pSHF populations; *TBX1* was preferentially expressed in aSHF progenitors, and *TBX5* was selectively enriched in pSHF progenitors (Fig. 1d), confirming successful regional specification under our conditions. We then asked whether *CHD7* loss alters these regional progenitor programs during subsequent patterning. RT–qPCR at D 3.5 and D 5.5 showed that *GATA4* expression was significantly reduced in LV (FHF-derived) lineages upon *CHD7* knockout (Fig. 1e). In the aSHF-associated trajectories, *TBX1* expression increased from D 3.5 to D 5.5 in RV and OFT in wild-type organoids, whereas *CHD7* loss significantly suppressed *TBX1* in both regions (Fig. 1f, g). Similarly, *TBX5* expression was elevated at D 5.5 in ATR and AVC lineages in wild-type organoids, but was significantly reduced in *CHD*7^⁻/⁻^ ATR and AVC organoids (Fig. 1h, i). These results indicate that CHD7 supports robust activation of region-appropriate progenitor gene programs during early patterning, prior to overt morphological divergence.

We subsequently generated the five chamber-like organoid subtypes from wild-type and *CHD7*^⁻/⁻^ hESCs (Fig. 1j). Wild-type organoids progressively self-organized into three-dimensional structures with enclosed cavity- or chamber-like architectures beginning around D3.5, consistent with early morphogenetic events of heart development. Across time points (days 1.5, 3.5, 5.5, 7.5, and 9.5), *CHD7*^⁻/⁻^ LV organoids were consistently smaller than their wild-type counterparts (Fig. 1k). RV organoids showed comparable size at early stages but became markedly reduced by D 9.5 (Fig. 1l). In addition, *CHD7*^⁻/⁻^ organoids displayed abnormal morphology most prominently in OFT and ATR lineages, where disorganization and size reduction became evident from D 5.5 onwards (Fig. 1m, n). By contrast, AVC organoids did not exhibit overt morphological abnormalities over the same differentiation period (Fig. 1o). Collectively, these findings show that CHD7 loss disrupts chamber morphogenesis in a region-dependent manner, with pronounced effects on growth and structural organization in specific chamber-like lineages during differentiation.

### Loss of CHD7 Preserves Pluripotency but Disrupts Chamber-Specific Differentiation

**Fig. 2:**
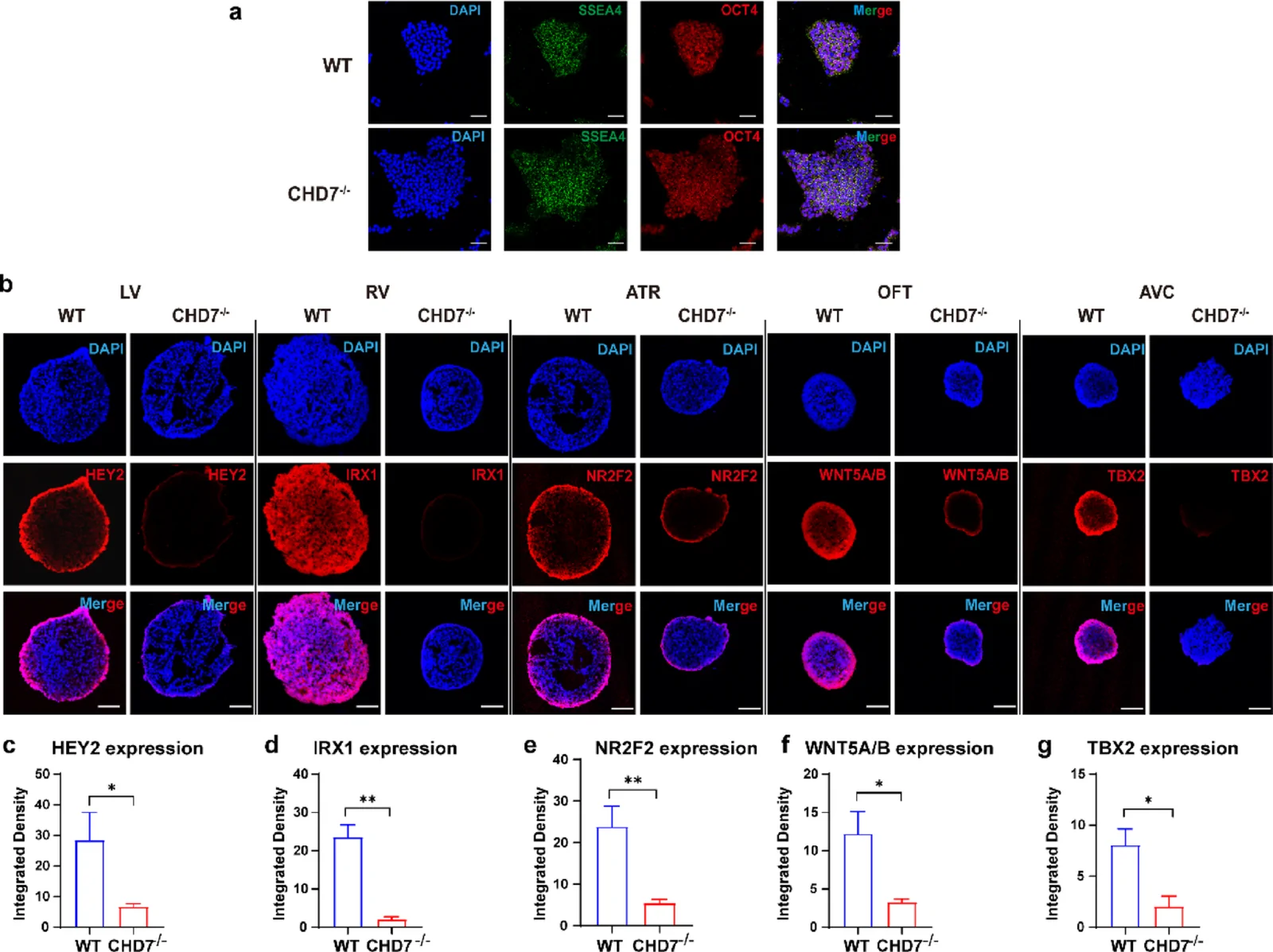
CHD7 knockout does not affect pluripotency but impairs cardiac lineage specification. (**a**) Immunostaining of pluripotent markers (OCT4, SSEA4) in cardiac organoids (*n *= 5), scale bars = 100 μm. (**b**) Immunostaining of subtype-specific markers in cardiac organoids, scale bars = 100 μm. (**c-g**) Quantification of HEY2, IRX1, NR2F2, WNT5A/B, and TBX2 expression in multi-chamber cardiac organoids (*n* = 5). Data are shown as means ± SEM. ns *p* > 0.05; *p< 0.05, **p < 0.01, ***p < 0.001

Immunofluorescence staining for pluripotency markers OCT4 and SSEA4 confirmed that CHD7 deletion does not compromise the pluripotent state of hESCs (Fig. 2a). We next assessed chamber-specific patterning within multi-chamber cardiac organoids using subtype-enriched markers: HEY2 (LV), IRX1 (RV), NR2F2 (ATR), WNT5A/B (OFT) and TBX2 (AVC) (Fig. 2b). Compared with wild-type organoids, *CHD7*^⁻/⁻^ organoids exhibited significantly reduced expression of each subtype marker across the corresponding regions (Fig. 2c–g), indicating impaired establishment of chamber-associated transcriptional identities. Together, these data show that CHD7 is dispensable for pluripotency maintenance but required for robust regional differentiation within multi-chamber cardiac organoids.

To determine whether this phenotype reflects a cardiac-selective defect rather than a broad impairment of mesodermal differentiation, we evaluated non-cardiac mesodermal lineages within the same organoid system. Immunofluorescence revealed robust expression of the smooth muscle–associated marker ACTA2 across LV, RV, OFT, ATR, and AVC regions (Fig. S3a), and CHD7 knockout did not measurably alter ACTA2 distribution or signal intensity in any chamber-derived region (Fig. S3b–f). Consistently, RT–qPCR showed no significant changes in smooth muscle–associated markers (*ACTA2*,* MYH11*) across LV, RV, OFT, ATR, and AVC organoids (Fig. S3g, h). Expression of skeletal muscle–associated markers (*MYOG*,* MYOD1*) likewise remained unchanged following CHD7 deletion (Fig. S3i, j). These results indicate that CHD7 loss does not broadly disrupt mesodermal differentiation in this model, supporting a preferential requirement for CHD7 in cardiac progenitor programs and regional cardiac differentiation.

### Loss of CHD7 Disrupts Cardiomyocyte Formation and Functional Maturation

**Fig. 3:**
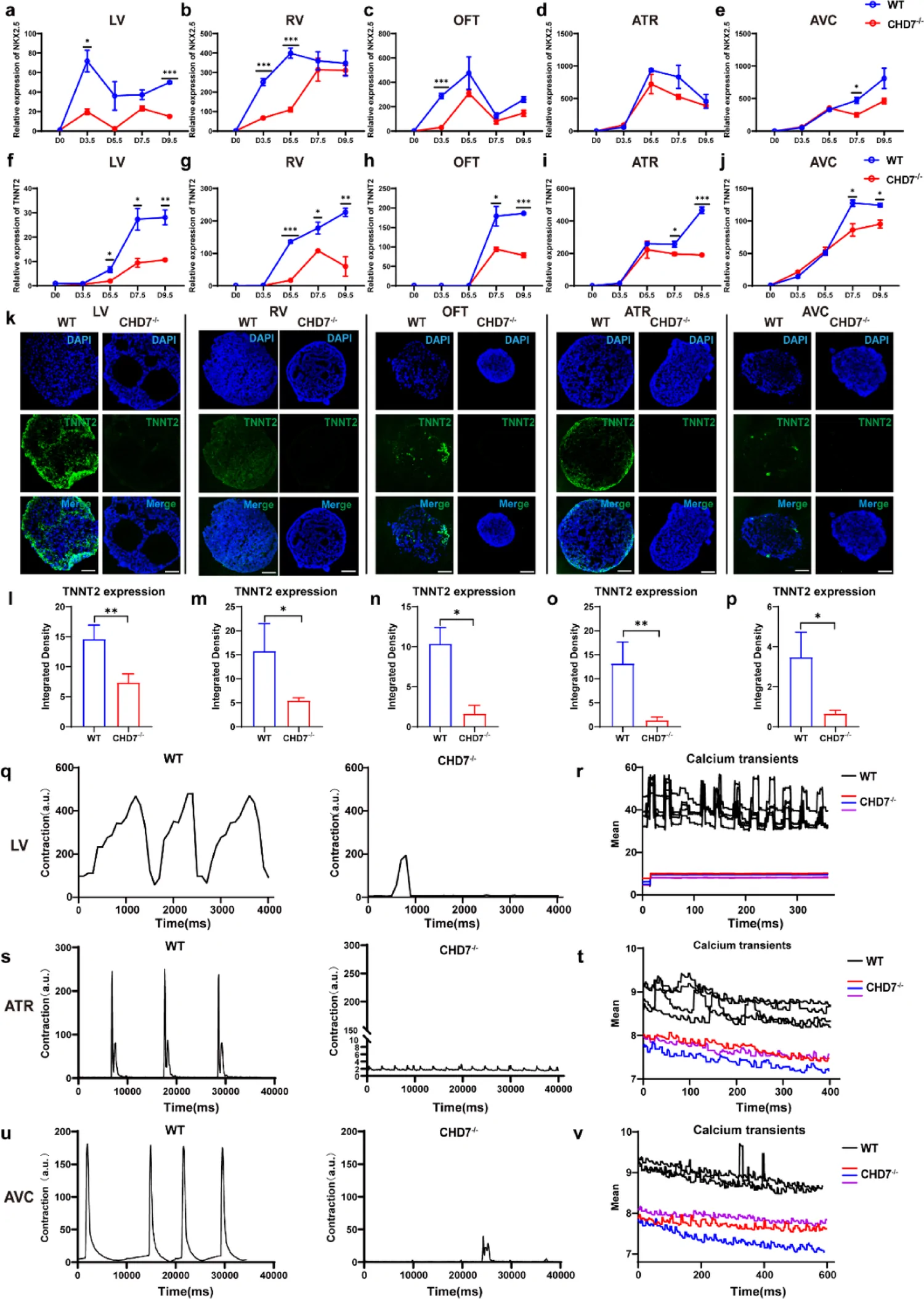
CHD7 knockout impairs cardiomyocyte identity and contractile function in cardiac organoids. (**a**-**e**) RT-qPCR showing the relative expression of *NKX2.5* in cardiac organoids from D0 to D9.5 (*n* = 3). (**f**-**j**) RT-qPCR showing the relative expression of *TNNT2* in cardiac organoids from D0 to D9.5 (*n* = 3). (**k**) Immunostaining of cardiomyocyte-specific markers (TNNT2) in cardiac organoids. (**l**-**p**) Quantification of TNNT2 expression in multi-chamber cardiac organoids (*n* = 5). (**q**) Contraction profiles of each individual beat generated using MUSCLEMOTION for WT and *CHD7*^−/−^ differentiated LV at D9.5 (*n* = 3). (**r**) Representative images of spontaneous calcium transients of WT and *CHD7*^−/−^ differentiated LV at D9.5 (*n* = 3), scale bars = 100 μm. (**s**) Contraction profiles of each individual beat generated using MUSCLEMOTION for WT and *CHD7*^−/−^ differentiated ATR at D9.5 (*n* = 3). (**t**) Representative images of spontaneous calcium transients of WT and *CHD7*^−/−^ differentiated ATR at D9.5 (*n* = 3). (**u**) Contraction profiles of each individual beat generated using MUSCLEMOTION for WT and *CHD7*^−/−^ differentiated AVC at D9.5 (*n* = 3). (**v**) Representative images of spontaneous calcium transients of WT and *CHD7*^−/−^ differentiated AVC at D9.5 (*n* = 3). Data are shown as means ± SEM. ns *pP* > 0.05; *p < 0.05, **p < 0.01, ***p < 0.001

To distinguish between a complete failure of cardiomyocyte lineage commitment and impaired differentiation upon CHD7 loss, we profiled the temporal expression of core cardiomyocyte markers across regionally specified organoids. RT–qPCR was performed at days 3.5, 5.5, 7.5, and 9.5 to quantify *NKX2.5* and *TNNT2* in LV, RV, OFT, ATR, and AVC lineages. In wild-type organoids, *NKX2.5* was robustly induced during the early progenitor window (D3.5–D5.5) and increased progressively through D9.5 across all regions (Fig. 3a–e), consistent with efficient establishment and maintenance of a cardiac progenitor program. In *CHD7*^⁻/⁻^ organoids, *NKX2.5* was also clearly induced in all regions, indicating that cardiomyocyte lineage commitment is initiated rather than abolished. However, *NKX2.5* levels were consistently reduced relative to wild-type, with a blunted and temporally delayed increase most evident at intermediate-to-late stages (days 5.5–9.5), suggesting impaired progenitor expansion or maintenance rather than an outright failure of lineage specification.

Similarly, *TNNT2* expression in wild-type organoids increased steadily over time across all regions (Fig. 3f–j), reflecting progressive cardiomyocyte differentiation and maturation. In contrast, CHD7-deficient organoids showed markedly attenuated *TNNT2* induction, characterized by reduced amplitude and delayed kinetics across LV, RV, OFT, ATR, and AVC lineages, with the most pronounced deficits at later stages. Thus, CHD7 loss primarily compromises differentiation efficiency and maturation downstream of initial lineage commitment.

Consistent with these transcriptional findings, immunofluorescence staining confirmed the presence of TNNT2^+^ cardiomyocytes across all chamber-like organoid types, including RV and OFT (Fig. 3k). Notably, the overall abundance of cardiomyocytes was significantly reduced in *CHD7*^⁻/⁻^ organoids (Fig. 3k), supporting a requirement for CHD7 in efficient cardiomyocyte production across multiple cardiac subtypes.

We next assessed functional maturation by quantifying spontaneous contraction dynamics from video recordings analyzed using ImageJ/Fiji. Wild-type LV, ATR, and AVC organoids displayed regular and coordinated beating, whereas *CHD7*^⁻/⁻^ organoids exhibited weak, irregular or absent contractions (Fig. 3q, s, and u). Quantitative analysis further revealed significant reductions in contraction parameters—including contraction duration, contraction amplitude, time-to-peak, peak amplitude and peak-to-peak interval—in *CHD7*^⁻/⁻^ LV, ATR, and AVC organoids compared with wild-type controls (Fig. S4). In agreement with these defects, calcium imaging demonstrated that *CHD7*^⁻/⁻^ organoids exhibited markedly diminished and irregular spontaneous calcium transients relative to wild-type organoids (Fig. 3r, t, and v), consistent with impaired excitation–contraction coupling.

### Endothelial Lineage Differentiation Remains Largely Intact Upon CHD7 Loss

**Fig. 4:**
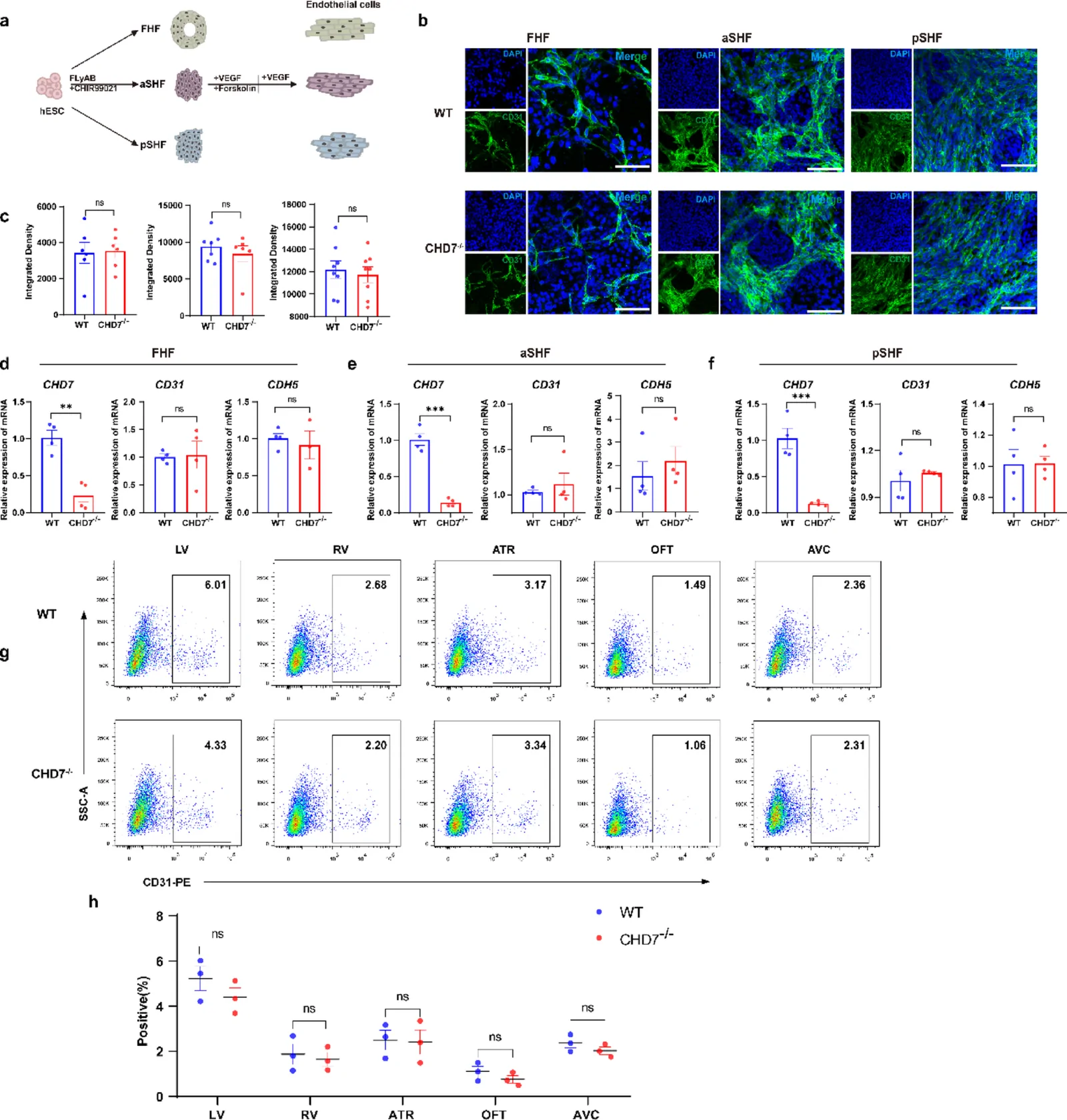
CHD7 knockout does not affect endothelial differentiation or cell proportion. (**a**) Schematic of the protocol for differentiation from hESCs to endothelial cells. (**b**) Immunostaining for CD31 in 2D endothelial cell differentiation, scale bars = 50 μm. (**c**) Quantification of CD31 in endothelial cells (*n* = 5). (**d**-**f**) RT-qPCR showing the relative expression of *CHD7*, *CD31*, and *CDH5* in 2D endothelial cell differentiation of all three progenitor populations (*n* = 3). (**g**-**h**) FACS quantification of CD31-positive cells in cardiac organoids (*n* = 3). Data are shown as means ± SEM. ns *pP* > 0.05; *pP < 0.05, **pP < 0.01, ***pP < 0.001

Our single-nucleus RNA-seq analysis (Fig. 1d–f) showed that CHD7 is broadly expressed across cardiac cell types, including cardiomyocytes and endothelial cells, raising the possibility that CHD7 may contribute to multiple lineages. To directly test whether CHD7 is required for endothelial differentiation, we induced endothelial lineage differentiation under 2D conditions using lineage-guided protocols corresponding to FHF- and SHF-associated trajectories (Fig. 4a).

Immunofluorescence staining for the endothelial marker CD31 (PECAM1) revealed no overt differences in endothelial morphology or CD31 signal between wild-type and *CHD7*^⁻/⁻^ cultures (Fig. 4b, c). Consistently, RT–qPCR confirmed marked reduction of CHD7 transcripts in the knockout group, whereas expression of endothelial markers *CDH5* (VE-cadherin) and *CD31* remained largely unchanged (Fig. 4d–f). We further quantified endothelial output by flow cytometry across chamber-derived organoid subtypes and found that CHD7 loss did not substantially alter the fraction of CD31^+^ cells in any subtype. Specifically, CD31^+^ cells comprised ~ 5% in LV, ~ 2% in RV, ~ 3% in ATR, ~ 1.5% in OFT, and ~ 2% in AVC organoids, comparable to wild-type controls (Fig. 4g, h). Together, these data indicate that endothelial differentiation efficiency is largely preserved in this system following CHD7 deletion.

### CHD7 Loss Suppresses Cardiomyocyte Structural and Calcium-handling Gene Programs in LV and ATR Organoids

**Fig. 5:**
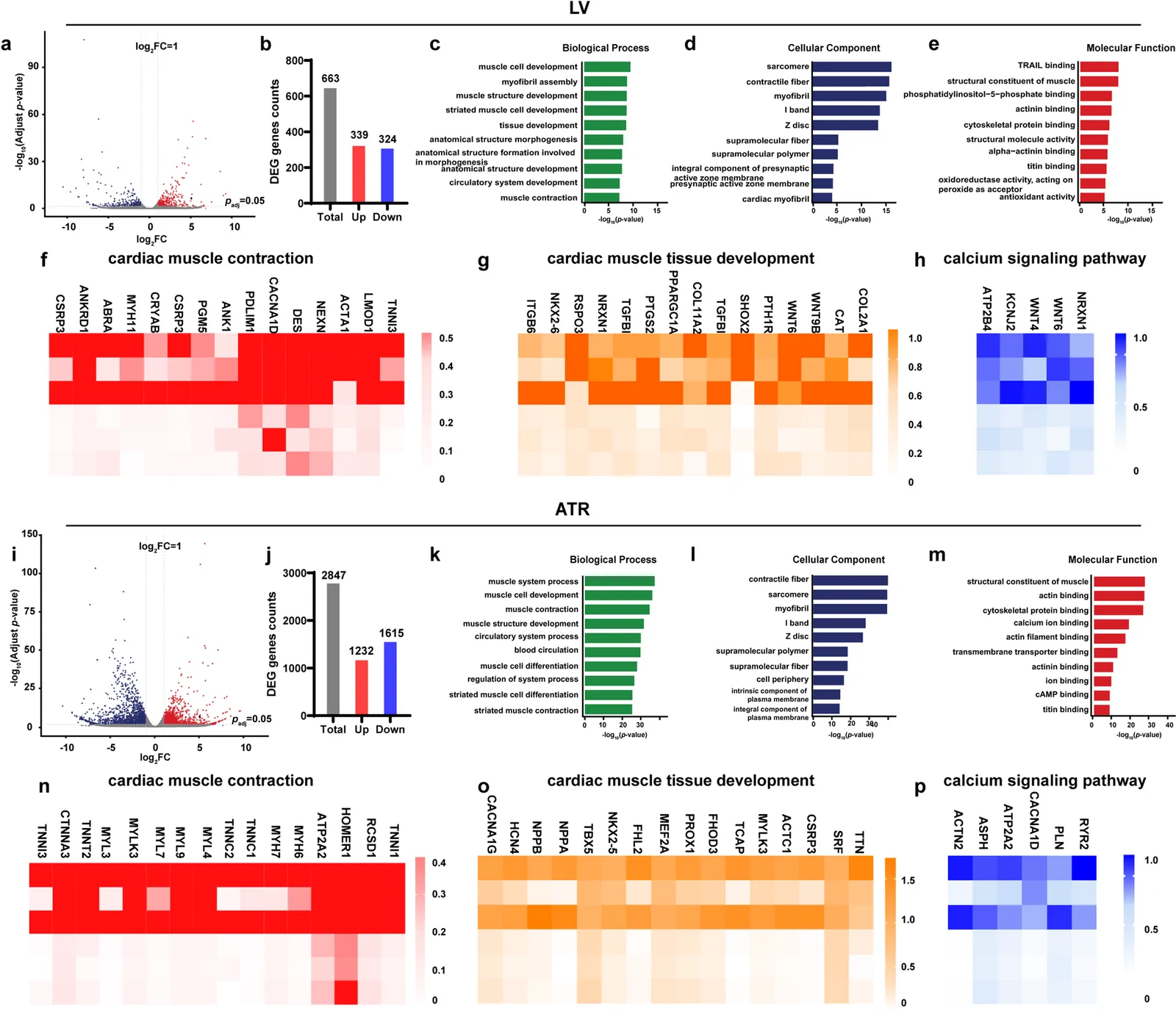
biological process, cellular component, and molecular function

**Fig. 6:**
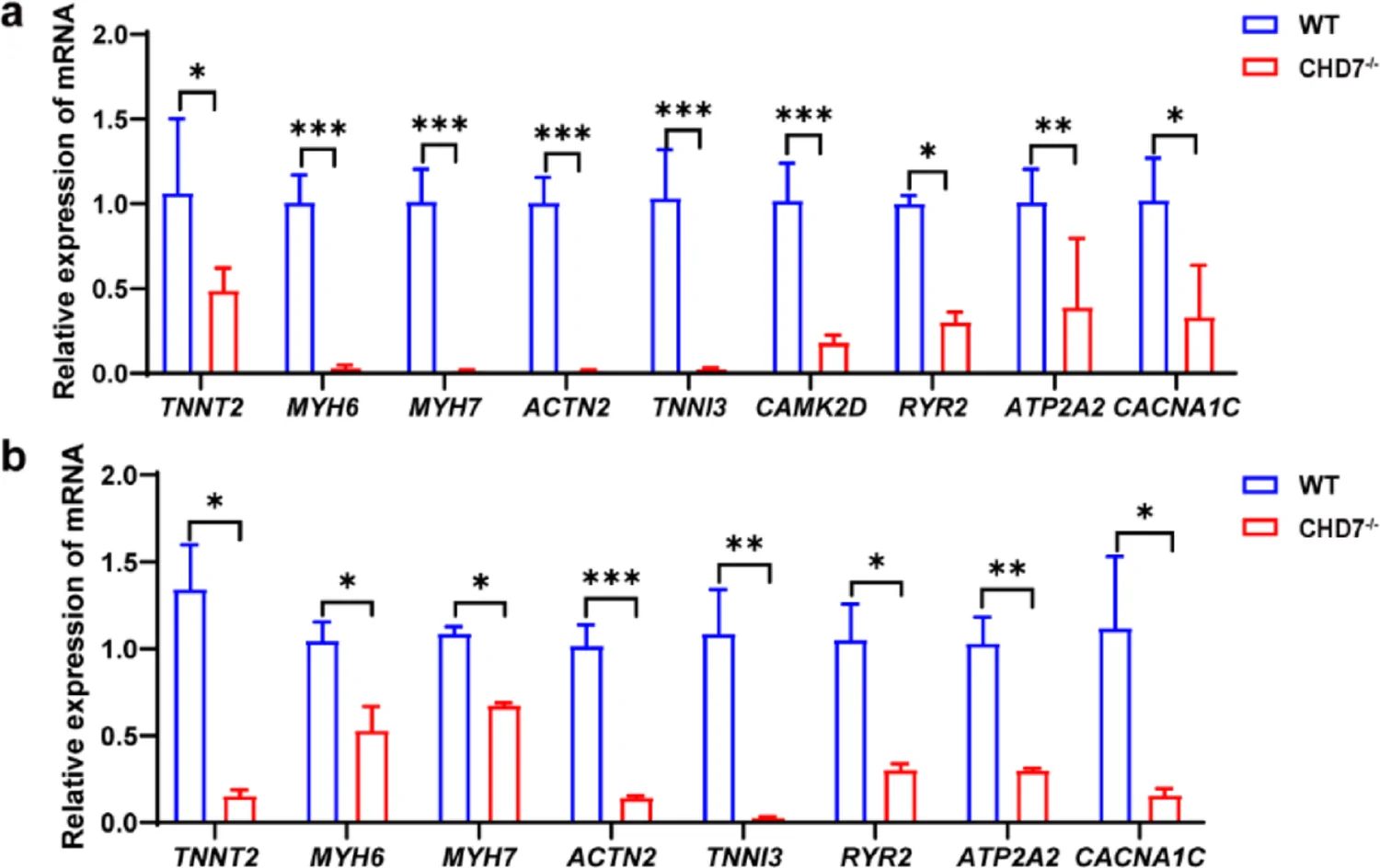
CHD7 knockout leads to downregulation of cardiomyocyte and calcium-related genes. (**a**) RT-qPCR showing the relative expression of *TNNT2*, *MYH6*, *MYH7*, *ACTN2*, *TNNI3*, *CAMK2D*, *RYR2*, *ATP2A2,* and *CACNA1C* in WT and *CHD7*^−/−^ differentiated LV at D9.5 (*n* = 3). (**b**) RT-qPCR showing the relative expression of *TNNT2*, *MYH6*, *MYH7*, *ACTN2*, *TNNI3*, *RYR2*, *ATP2A2,* and *CACNA1C* in WT and *CHD7*^−/−^ differentiated ATR at D9.5 (*n* = 3). Data are shown as means ± SEM. ns *p* > 0.05; *p < 0.05, **p < 0.01, ***p < 0.001

Given the pronounced morphological and contractile phenotypes observed in LV and ATR organoids, we performed bulk RNA-seq at D9.5 to define transcriptional consequences of CHD7 loss and to identify affected biological pathways. In LV organoids, differential expression analysis identified 339 significantly upregulated and 324 significantly downregulated genes, visualized by volcano plots (Fig. 5a, b). Gene Ontology (GO) enrichment analysis of these DEGs highlighted strong perturbation of muscle-related biological processes, including muscle cell development, myofibril assembly, muscle structure development, and striated muscle cell development (Fig. 5c). Cellular component terms were enriched for contractile structures such as sarcomere, contractile fibre, myofibril, I band, and Z disc (Fig. 5d), and molecular function analysis showed prominent enrichment for structural constituent of muscle (Fig. 5e). Consistent with these pathway-level changes, heatmaps visualized coordinated dysregulation of representative genes within these contractile and myofibrillar programs (Fig.5 f–h). RT–qPCR validation confirmed significantly reduced expression of key myofibril/sarcomere genes in LV organoids, including *TNNT2*, *MYH6*, *MYH7*, *ACTN2*, and *TNNI3*, together with downregulation of calcium-handling–contraction coupling genes *CAMK2D*, *RYR2*, A*TP2A2*, and *CACNA1C* (Fig. 6a).

A similar transcriptional signature was observed in ATR organoids. Volcano plots showed 1232 significantly upregulated and 1615 significantly downregulated genes in *CHD7*^⁻/⁻^ ATR organoids compared with controls (Fig. 5i, j). GO enrichment again prioritized muscle-related biological processes, including muscle system process, muscle cell development, muscle contraction, and muscle structure development (Fig. 5k). Cellular component terms were enriched for contractile fibre, sarcomere, myofibril, I band, and Z disc (Fig. 5l), while molecular function terms were again dominated by structural constituent of muscle (Fig. 5m). Heatmaps further highlighted coherent dysregulation of genes within these muscle and contractile programs (Fig. 5n–p). RT–qPCR validation confirmed significant downregulation of key myofibrillar genes (*TNNT2*, *MYH6*, *MYH7*, *ACTN2*, and *TNNI3*) and reduced expression of calcium-handling genes including *RYR2*, *ATP2A2*, and *CACNA1C* in ATR organoids (Fig. 6b). Collectively, these data indicate that CHD7 loss broadly suppresses cardiomyocyte structural and excitation–contraction coupling gene programs in chamber-like organoids, consistent with impaired cardiomyocyte maturation and function.

## Discussion

CHD7 is an ATP-dependent, chromatin-associated remodeler that is indispensable for embryonic development and has been implicated in diverse organ systems, including neurogenesis (Gao et al. 2024; Huang et al. 2025; Kim and Roberts 2013), inner ear formation (Nie et al. 2022; Yao et al. 2018), ocular system (Krueger and Morris 2022), genital system (Antoniou et al. 2019; Dauber et al. 2010), and skeletal system development (Breuer et al. 2024; Liu et al. 2022). Despite its broad developmental expression and the multisystem nature of CHARGE syndrome, a cardiac-focused investigation is strongly justified (Bergman et al. 2011; Jyonouchi et al. 2009; van Ravenswaaij-Arts and Martin 2017). Pathogenic *CHD7* variants account for the majority of CHARGE syndrome cases (reported in ~ 70% of affected individuals), and congenital heart disease is among the most prevalent and clinically consequential manifestations, often shaping early survival and long-term outcomes (Bergman et al. 2011; Janssen et al. 2012; Jyonouchi et al. 2009). Consistent with this clinical burden, our integrative analyses of human and mouse developmental transcriptomic datasets show that CHD7 is expressed at relatively high and dynamic levels during early cardiogenesis, with peak expression coinciding with key windows of cardiac morphogenesis and chamber specification (Fig. S1a). Together, these observations support an important role for CHD7 in cardiomyocyte development and cardiac structural assembly.

Here, we used a three-dimensional, multi-chamber cardiac organoid model derived from hESCs to interrogate CHD7 function in human cardiogenesis and to model CHARGE-associated cardiac pathology *in vitro*. CHD7 loss disrupted organoid architecture, weakened chamber boundary definition and reduced the efficiency of cardiomyocyte differentiation. This was supported by concordant morphological and molecular phenotypes, including smaller organoids with blurred chamber borders and markedly reduced expression of cardiomyocyte structural genes (*TNNT2*, *MYH6*, *MYH7*, *ACTN2* and *TNNI3*) (Fig. 6). In contrast, endothelial differentiation was comparatively preserved: endothelial markers such as *CD31* and *CDH5* were largely unchanged (Fig. 4), and the proportion of CD31⁺ endothelial cells remained similar across chamber-derived subtypes. These lineage-biased phenotypes argue against a global impairment of cardiac cell fates and instead point to a preferential requirement for CHD7 in cardiomyocyte lineage progression and maturation.

Importantly, our data further indicate that CHD7 deficiency does not produce a generalized mesodermal differentiation defect. Supplementary analyses showed comparable ACTA2 signal across LV, RV, OFT, ATR, and AVC regions in control and *CHD7*^−/−^ organoids (Fig. S3a–f), and RT–qPCR revealed no significant changes in smooth muscle–associated markers *(ACTA2*,* MYH11*) or skeletal muscle–associated markers (*MYOG*,* MYOD1*) across these chamber-derived lineages (Fig. S3g–j). Together, these results support the conclusion that the dominant consequence of CHD7 loss in this system is a selective impairment of cardiomyocyte differentiation efficiency and maturation rather than a broad blockade of mesodermal lineage specification.

Functionally, *CHD7*^−/−^ organoids exhibited weaker and less synchronized calcium transients with reduced frequency and amplitude, consistent with impaired contractility and electrophysiological competence. In agreement with these physiological deficits, RT–qPCR validated downregulation of core excitation–contraction coupling and calcium-handling genes, including *RYR2*, *CACNA1C*, *ATP2A2*, *CACNA2D,* and *TNNT2* (Fig. 6). Together with the coordinated suppression of sarcomere-related programs observed in our transcriptomic analyses, these findings indicate that CHD7 is required not only for efficient cardiomyocyte production but also for their structural and functional maturation, likely through maintaining contractile and calcium-handling gene programs.

Mechanistically, although our RNA-seq data reveal robust suppression of sarcomere and calcium-handling modules, transcriptomic changes alone do not establish direct CHD7 occupancy at each affected locus. A plausible framework is enhancer-centric regulation: CHD7 has been reported to occupy dynamically regulated cardiac enhancers and to physically interact with the cardiac pioneer factor ISL1, co-occupying ISL1-regulated enhancer elements to control heart-field gene expression during morphogenesis (Stathopoulou et al. 2023). Such enhancer-associated remodeling provides a coherent explanation for how reduced CHD7 activity could weaken upstream developmental circuits and propagate to downstream structural and electrophysiological programs, including sarcomere assembly and excitation–contraction coupling. Future CHD7 CUT&Tag/ChIP-seq and transcription factor co-occupancy analyses in the organoid context will be important to distinguish direct targets from secondary consequences of altered developmental state.

The organoid findings are also consistent with clinical observations in CHARGE syndrome, in which congenital heart defects such as ventricular hypoplasia and atrioventricular septal defects are frequently reported (Bergman et al. 2011; Gug et al. 2020; Huang et al. 2023; Stathopoulou et al. 2023; van Ravenswaaij-Arts and Martin 2017; Vissers et al. 2004; Wang et al. 2022, 2025; Yan et al. 2020; Zhang et al. 2024). The phenotypic similarity between *CHD7*^−/−^ multi-chamber organoids and CHARGE-associated cardiac malformations supports the view that CHD7 insufficiency contributes directly to at least a subset of cardiac developmental abnormalities. This interpretation is further supported by *in vivo* genetic evidence: cardiogenic mesoderm-restricted deletion of Chd7 using *Mesp1*-cre leads to outflow tract malformations, intracardiac septation defects, and impaired cardiomyocyte maturation with compromised excitation–contraction coupling, paralleling the developmental and functional gene programs disrupted in our CHD7-deficient organoids (Park et al. 2025; Stathopoulou et al. 2023). In addition, a CHD7 missense variant (T730 in human; T720 in mouse) has been linked to cardiomyopathy and a neonatal cardiac transcriptomic signature characterized by reduced expression of mitochondrial energy metabolism genes, supporting a dosage-sensitive requirement for CHD7 that couples early developmental gene regulation to later metabolic and contractile maturation (Park et al. 2025). At the same time, we acknowledge inherent limitations of organoid systems: multi-chamber cardiac organoids cannot fully capture *in vivo* inputs such as hemodynamic loading, neurohumoral regulation, biomechanical forces, vascular perfusion and innervation, all of which shape chamber morphogenesis and promote late-stage cardiomyocyte maturation. These constraints should be considered when interpreting maturation-related phenotypes and when aligning *in vitro* trajectories with *in vivo* development.

Finally, a key advantage of multi-chamber cardiac organoids is the ability to model lineage- and domain-restricted differentiation in a controlled human setting. By stage-specific modulation of Activin/Nodal, WNT, BMP, and retinoic acid signaling, this platform generates organoid domains corresponding to major developmental lineages, including FHF-, aSHF- and pSHF-associated territories (Schmidt et al. 2023). This enables mechanistic interrogation of gene function across distinct progenitor compartments while preserving higher-order morphogenetic features. Collectively, our study identifies CHD7 as a crucial regulator of efficient cardiomyocyte differentiation and functional maturation and provides mechanistic insight into CHARGE syndrome–associated congenital heart defects; more broadly, delineation of the CHD7-centred regulatory network may inform future efforts to mitigate developmental perturbations that culminate in congenital heart disease.

## Conclusions

Here, we use a multi-chamber cardiac organoid model to establish a molecular link between human cardiogenesis and CHD7-dependent chromatin control. We demonstrate that CHD7 is necessary for both robust cardiac progenitor progression and the effective structural and functional maturation of cardiomyocytes, rather than serving as an absolute determinant of lineage commitment, by combining functional phenotyping with genetic perturbation and molecular profiling. These results highlight CHD7-centered regulatory programs as possible candidates for further mechanistic and translational research and offer a framework for comprehending how CHD7 deficiency may lead to congenital cardiac abnormalities in CHARGE syndrome.

## Electronic Supplementary Material

Below is the link to the electronic supplementary material.

Supplementary Material 1

## Data Availability

The data generated in this study are available upon request from the corresponding author.

## Abbreviations

ACTN2: Alpha actinin 2
aSHF: Anterior second heart field
ATP2A2: ATPase sarcoplasmic/endoplasmic reticulum Ca^2+^ transporting 2
CACNA1C: Calcium voltage-gated channel subunit alpha1 C
CAMK2D: Calcium/calmodulin dependent protein kinase II delta
CHD7: Chromodomain helicase DNA-binding protein 7
CHARGE syndrome: Coloboma, Heart defects, Atresia choanae, Retarded growth and development, Genital abnormalities, Ear abnormalities syndrome
FGF2: Fibroblast growth factor 2
FHF: First heart field
hESC: Human embryonic stem cell
MYH6: Myosin heavy chain 6
MYH7: Myosin heavy chain 7
pSHF: Posterior second heart field
RYR2: Ryanodine receptor 2
TNNT2: Troponin T2
VEGF: Vascular endothelial growth factor

## References

[CR1] Althali NJ, Hentges KE (2022) Genetic insights into non-syndromic tetralogy of fallot. Front Physiol 13:1012665 10.3389/fphys.2022.1012665 PMC9582763 36277185

[CR2] Antoniou MC, Bouthors T, Xu C et al (2019 )A novel CHD7 mutation in an adolescent presenting with growth and pubertal delay. Ann Pediatr Endocrinol Metab 24, 49–54 10.6065/apem.2019.24.1.49 PMC6449621 30943680

[CR3] Bergman JE, Janssen N, Hoefsloot LHet al (2011) CHD7 mutations and CHARGE syndrome: the clinical implications of an expanding phenotype. J Med Genet 48:334–342 10.1136/jmg.2010.087106 21378379

[CR4] Breuer M, Rummler M, Singh J et al (2024) CHD7 regulates craniofacial cartilage development via controlling HTR2B expression. J Bone Min Res 39:498–512 10.1093/jbmr/zjae024 PMC11262153 38477756

[CR5] Dauber A, Hirschhorn JN, Picker J et al (2010) Delayed puberty due to a novel mutation in CHD7 causing CHARGE syndrome. Pediatrics 126:e1594–1598 10.1542/peds.2010-0164 21041284

[CR6] Feng Z, Huang X, Gao Y et al (2024) MST1R gene variants predispose individuals to tetralogy of fallot. Phenomics 4:548–561 10.1007/s43657-024-00175-9 PMC11889330 40061823

[CR7] Gao J, Skidmore JM, Cimerman J et al (2024) CHD7 and SOX2 act in a common gene regulatory network during mammalian semicircular Canal and cochlear development. Proc Natl Acad Sci U S A 121:e2311720121 10.1073/pnas.2311720121 PMC10927591 38408234

[CR8] Gug C, Gorduza EV, Lacatusu A et al (2020) CHARGE syndrome associated with de Novo (I1460Rfs(*)15) frameshift mutation of CHD7 gene in a patient with arteria lusoria and horseshoe kidney. Exp Ther Med 20:479–485 10.3892/etm.2020.8683 PMC7271729 32509017

[CR9] Hofbauer P, Jahnel SM, Papai N et al (2021) Cardioids reveal self-organizing principles of human cardiogenesis. Cell 184:3299–3317e3222 10.1016/j.cell.2021.04.034 34019794

[CR10] Huang X, Gao H, Chen W et al (2023) Analysis of the CHD7 gene mutations in patients of congenital heart disease with extracardiac malformations. Transl Pediatr 12:1148–1160 10.21037/tp-22-634 PMC10326764 37427070

[CR11] Huang Z, He C, Wang G et al (2025) Mutation of CHD7 impairs the output of neuroepithelium transition that is reversed by the Inhibition of EZH2. Mol Psychiatry 30:4094–4109 10.1038/s41380-025-02990-6 40164694

[CR12] Hurd EA, Capers PL, Blauwkamp MN et al (2007) Loss of Chd7 function in gene-trapped reporter mice is embryonic lethal and associated with severe defects in multiple developing tissues. Mamm Genome 18:94–104 10.1007/s00335-006-0107-6 17334657

[CR13] Issekutz KA, Graham JJ, Prasad C et al (2005) An epidemiological analysis of CHARGE syndrome: preliminary results from a Canadian study. Am J Med Genet A 133A:309–317 10.1002/ajmg.a.30560 15637722

[CR14] Jamadagni P, Breuer M, Schmeisser K et al (2021) Chromatin remodeller CHD7 is required for GABAergic neuron development by promoting PAQR3 expression. EMBO Rep 22:e50958 10.15252/embr.202050958 PMC8183419 33900016

[CR15] Janssen N, Bergman JE, Swertz MA et al (2012) Mutation update on the CHD7 gene involved in CHARGE syndrome. Hum Mutat 33:1149–1160 10.1002/humu.22086 22461308

[CR16] Jyonouchi S, McDonald-McGinn DM, Bale S et al(2009) CHARGE (coloboma, heart defect, Atresia choanae, retarded growth and development, genital hypoplasia, ear anomalies/deafness) syndrome and chromosome 22q11.2 deletion syndrome: a comparison of Immunologic and nonimmunologic phenotypic features. Pediatrics 123:e871–877 10.1542/peds.2008-3400 PMC4098848 19403480

[CR18] Kim KH, Roberts CW (2013) CHD7 in charge of neurogenesis. Cell Stem Cell 13:1–2 10.1016/j.stem.2013.06.010 23827700

[CR17] Kim H, Kamm RD, Vunjak-Novakovic G et al(2022) Progress in multicellular human cardiac organoids for clinical applications. Cell Stem Cell 29:503–514 10.1016/j.stem.2022.03.012 PMC9352318 35395186

[CR19] Krueger LA, Morris AC (2022) Eyes on CHARGE syndrome: roles of CHD7 in ocular development. Front Cell Dev Biol 10:994412 10.3389/fcell.2022.994412 PMC9512043 36172288

[CR20] Lewis-Israeli YR, Wasserman AH, Gabalski MA et al(2021) Self-assembling human heart organoids for the modeling of cardiac development and congenital heart disease. Nat Commun 12:5142 10.1038/s41467-021-25329-5 PMC8390749 34446706

[CR21] Liu C, Xiong Q, Li Q et al(2022) CHD7 regulates bone-fat balance by suppressing PPAR-gamma signaling. Nat Commun 13, 1989 10.1038/s41467-022-29633-6 PMC9007978 35418650

[CR22] Love MI, Huber W, Anders S (2014) Moderated Estimation of fold change and dispersion for RNA-seq data with DESeq2. Genome Biol 15:550 10.1186/s13059-014-0550-8 PMC4302049 25516281

[CR23] Mendjan S, Deyett A, Yelon D (2025) Coordination of cardiogenesis in vivo and in vitro. Nat Rev Mol Cell Biol 27(1):19-34 10.1038/s41580-025-00878-5 40993223

[CR24] Nie J, Ueda Y, Solivais AJ et al(2022) CHD7 regulates otic lineage specification and hair cell differentiation in human inner ear organoids. Nat Commun 13:7053 10.1038/s41467-022-34759-8 PMC9672366 36396635

[CR25] Park IY, Hsu CW, Bouazoune K et al(2025) A novel cardiomyopathy phenotype linked to a CHD7 missense variant. Sci Rep 15:19429 10.1038/s41598-025-00606-1 PMC12134341 40461563

[CR26] Patel L, Roy A, Barlow J et al (2024) Characterisation of infantile cardiomyopathy in Alstrom syndrome using ALMS1 knockout induced pluripotent stem cell derived cardiomyocyte model. Mol Genet Metab 143:108575 10.1016/j.ymgme.2024.108575 39243575

[CR27] Sala L, van Meer BJ, Tertoolen LGJ et al(2018) MUSCLEMOTION: A versatile open software tool to quantify cardiomyocyte and cardiac muscle contraction in vitro and in vivo. Circ Res 122:e5–e16 10.1161/CIRCRESAHA.117.312067 PMC5805275 29282212

[CR28] Schmidt C, Deyett A, Ilmer T et al(2023) Multi-chamber cardioids unravel human heart development and cardiac defects. Cell 186:5587–5605e5527 10.1016/j.cell.2023.10.030 38029745

[CR29] Srivastava D (2021) Modeling human cardiac chambers with organoids. N Engl J Med 385:847–849 10.1056/NEJMcibr2108627 34437788

[CR30] Stathopoulou A, Wang P, Thellier C et al(2023) CHARGE syndrome-associated CHD7 acts at ISL1-regulated enhancers to modulate second heart field gene expression. Cardiovasc Res 119:2089–2105 10.1093/cvr/cvad059 PMC10478754 37052590

[CR31] Trapnell C, Pachter L, Salzberg SL (2009) TopHat: discovering splice junctions with RNA-Seq. Bioinformatics 25:1105–1111 10.1093/bioinformatics/btp120 PMC2672628 19289445

[CR32] van Ravenswaaij-Arts C, Martin DM (2017) New insights and advances in CHARGE syndrome: Diagnosis, etiologies, treatments, and research discoveries. Am J Med Genet C Semin Med Genet 175:397–406 10.1002/ajmg.c.31592 PMC6591023 29171162

[CR33] Vissers LE, van Ravenswaaij CM, Admiraal R et al (2004) Mutations in a new member of the chromodomain gene family cause CHARGE syndrome. Nat Genet 36:955–957 10.1038/ng1407 15300250

[CR35] Wang S, Lin Y, Liang P et al (2022) De Novo splice site mutation of the CHD7 gene in a Chinese patient with typical CHARGE syndrome. ORL J Otorhinolaryngol Relat Spec 84:417–424 10.1159/000520376 35078197

[CR34] Wang G, Huang Z, He C et al (2025) ATPase-deficient CHD7 disease variant disrupts neural development via chromatin dysregulation. J Genet Genomics52(10):1268-1282 10.1016/j.jgg.2025.08.012 40915599

[CR36] Yan S, Thienthanasit R, Chen D et al (2020) CHD7 regulates cardiovascular development through ATP-dependent and -independent activities. Proc Natl Acad Sci U S A 117:28847–28858 10.1073/pnas.2005222117 PMC7682373 33127760

[CR37] Yao H, Hill SF, Skidmore JM et al(2018) CHD7 represses the retinoic acid synthesis enzyme ALDH1A3 during inner ear development. JCI Insight 3 (4):e97400 10.1172/jci.insight.97440 PMC5916250 29467333

[CR38] Zhang Y, Lu Y, Long X et al (2024) A case report of CHARGE syndrome caused by a de Novo CHD7 gene mutation. SAGE Open Med Case Rep 12:2050313X241293307 12: 1 –7 10.1177/2050313X241293307 PMC11607752 39618705

