# Supplementary material for: Cardiac Organoids Uncover an Association of CHD7 Deficiency with Congenital Heart Disease
Source: Phenomics. 2026 Jul 9;6(5):652–67. doi: 10.1007/s43657-026-00319-z (PMC13743710; doi:10.1007/s43657-026-00319-z)
Supplement: Supplementary file 1 [file 43657_2026_319_MOESM1_ESM.docx]

**Supplementary Figure1.Expression pattern of CHD7 in human fetal hearts and mouse hearts.** Relative mRNA expression levels of *CHD7* across various human fetal tissues using the GSE64283 dataset. (b) Temporal expression profile of *CHD7* in 10-week human fetal hearts based on the GSE64283 dataset. (c) Dynamic expression of *Chd7* during mouse heart development from E7.5 to adulthood, based on GSE51483. (d–e) UMAP plots showing the distribution and expression of CHD7 across different cell types in the human fetal heart based on the GSE156707. (f) Quantification of CHD7 expression levels in individual cardiac cell types using the GSE156707 single-nucleus RNA-seq dataset.

 **Supplementary Figure2.** (a) CHD7 immunostaining in cardiac organoids.

**Supplementary Figure3.** (a) ACTA2 immunostaining in cardiac organoids. (b-f) Quantification of ACTA2 expression in multi-chamber cardiac organoids (n = 3). (g-j) RT-qPCR showing the relative expression of *ACTA2*, *MYH11*, *MYOG* and *MYOD1* in cardiac organoids (n = 3). Data are shown as means ± SEM. ns *P* > 0.05; **P* < 0.05, ***P* < 0.01, ****P* < 0.001.

**Supplementary Figure4.** **Quantification of beating activity in multi-chamber cardiac organoids after *CHD7* knockout.** (a-e) Quantitative analysis of contraction duration, contraction amplitude, Time-to peak, Peak amplitude and Peak-to-peak time of LV (n =3). (f-j) Quantitative analysis of contraction duration, contraction amplitude, Time-to peak, Peak amplitude and Peak-to-peak time of ATR (n =3). (k-o) Quantitative analysis of contraction duration, contraction amplitude, Time-to peak, Peak amplitude and Peak-to-peak time of AVC (n=3). Data are shown as means ± SEM. ns *P* > 0.05; **P* < 0.05, ***P* < 0.01, ****P* < 0.001.

**Supplementary Table 1. Primers sequences used for qRT-PCR**

| Gene | | Forward Primer | | Reverse Primer |
| --- | --- | --- | --- | --- |
| *GAPDH* | 5′- GTCTCCTCTGACTTCAACAGCG-3′ | | 5′- ACCACCCTGTTGCTGTAGCCAA-3′ | |
| *TNNT2* | 5′- AAGAGGCAGACTGAGCGGGAAA-3′ | | 5′- AGATGCTCTGCCACAGCTCCTT-3′ | |
| *MYH6* | 5′- GGAAGACAAGGTCAACAGCCTG-3′ | | 5′- TCCAGTTTCCGCTTTGCTCGCT-3′ | |
| *MYH7* | 5′- GGAGTTCACACGCCTCAAAGAG-3′ | | 5′- TCCTCAGCATCTGCCAGGTTGT-3′ | |
| *ACTN2* | 5′-GAGGGCAAGATGGTGTCGGATA-3′ | | 5′-CTTCTCAGCCAGGTGTTCCAAG-3′ | |
| *TNNI3* | 5′-CGTGTGGACAAGGTGGATGAAG-3′ | | 5′-GCCGCTTAAACTTGCCTCGAAG-3′ | |
| *CAMK2D* | 5′-ACACGGTGACTCCTGAAGCCAA-3′ | | 5′-GTCTCCTGTGTGTGCATCATGG-3′ | |
| *RYR2* | 5′- CTTGAGGTTGGCTTTCTGCCAG-3′ | | 5′- TGTGCCAGCAAAGAGAGGAGCA-3′ | |
| *ATP2A2* | 5′-GGACTTTGAAGGCGTGGATTGTG-3′ | | 5′-CTCAGCAAGGACTGGTTTTCGG-3′ | |
| *CACNA1C* | 5′-GCAGGAGTACAAGAACTGTGAGC-3′ | | 5′-CGAAGTAGGTGGAGTTGACCAC-3′ | |
| *NKX2.5* | 5′-AAGTGTGCGTCTGCCTTTCCCG-3′ | | 5′-TTGTCCGCCTCTGTCTTCTCCA-3′ | |
| *TBX1* | 5′-CGCAGTGGATGAAGCAAATCGTG-3′ | | 5′-TTTGCGTGGGTCCACATAGACC-3′ | |
| *TBX5* | 5′-ACAACCTGGACCCATTTGG-3′ | | 5′-GGAAAGACGTGAGTGCAGAACG-3′ | |
| *GATA4* | 5′-GCGGTGCTTCCAGCAACTCCA-3′ | | 5′-GACATCGCACTGACTGAGAACG-3′ | |
| *ACTA2* | 5′-CTATGCCTCTGGACGCACAACT-3′ | | 5′-CAGATCCAGACGCATGATGGCA-3′ | |
| *MYH11* | 5′-GTCCAGGAGATGAGGCAGAAAC-3′ | | 5′-GTCTGCGTTCTCTTTCTCCAGC-3′ | |
| *MYOD1* | 5′-CTCCAACTGCTCCGACGGCAT-3′ | | 5′-ACAGGCAGTCTAGGCTCGACAC-3′ | |
| *MYOG* | 5′-ACAGGCAGTCTAGGCTCGACAC-3′ | | 5′-AGGCGCTGTGAGAGCTGCATTC-3′ | |
